# Investigations Into the Metabolism and Elimination of Tesofensine in Human Urine

**DOI:** 10.1002/dta.70104

**Published:** 2026-06-19

**Authors:** O. Krug, A. Thomas, M. Thevis

**Affiliations:** ^1^ Institute of Biochemistry, Center for Preventive Doping Research (ZePräDo) German Sport University Cologne Cologne Germany; ^2^ European Monitoring Center for Emerging Doping Agents (EuMoCEDA) Cologne/Bonn Germany

**Keywords:** in vitro, in vivo, LC–MS, metabolism, tesofensine, urine

## Abstract

Tesofensine (NS‐2330) is a pharmacologically active compound with weight‐reducing effects in obese patients. Although still under regulatory review, it has been marketed online as a dietary supplement promoted for weight management and metabolic enhancement. Due to its impact on body weight, tesofensine could be relevant in competitive sports, particularly in weight‐class disciplines and sports where power‐to‐weight ratio is decisive. It is classified under “S6 stimulants” on the World Anti‐Doping Agency's Prohibited List and is prohibited in‐competition only, making detailed knowledge of its metabolism and excretion essential for anti‐doping purposes. Although the pharmacological effects and elimination of tesofensine and one dealkylated metabolite were described previously, elimination profiles and structural information on additional metabolites have been limited. In this study, in vitro metabolism experiments were conducted, followed by investigation of urinary metabolism and elimination in six volunteers after ingestion of 483 μg tesofensine as a dietary supplement. Urine was collected for up to 600 h, prepared by solid‐phase extraction, and analyzed by LC‐HRMS. Four principal metabolites were identified: three dealkylated metabolites (M1–M3) and one hydroxylated and glucuronidated metabolite (M4), supported by MS/MS dissociation patterns. The validated analytical method for human urine showed an LOD of 0.01 ng/mL, 34% recovery, and 8% interday imprecision. Marked interindividual variability was observed, with peak concentrations of 1–4 ng/mL after 4–46 h and detection windows up to 500 h. The findings enhance analytical procedures and suggest that recommended dosing is unlikely to result in concentrations constituting an Adverse Analytical Finding (AAF) under currently applicable stimulant minimum reporting levels.

## Graphical abstract

This study investigated tesofensine metabolism and urinary elimination after a single 483 μg intake via a dietary supplement. In six volunteers, urine was collected up to 659 h. LC‐HRMS/MS identified four tentative metabolites; maximum urinary concentrations reached 4 ng/mL within 4–48 h.

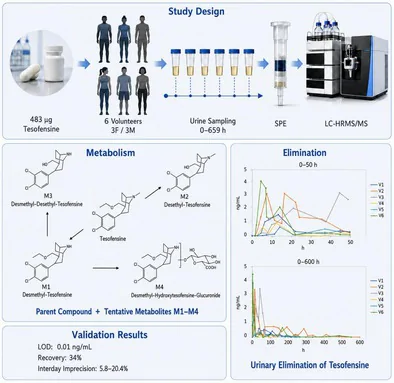

## 1 Introduction

**FIGURE 1:**
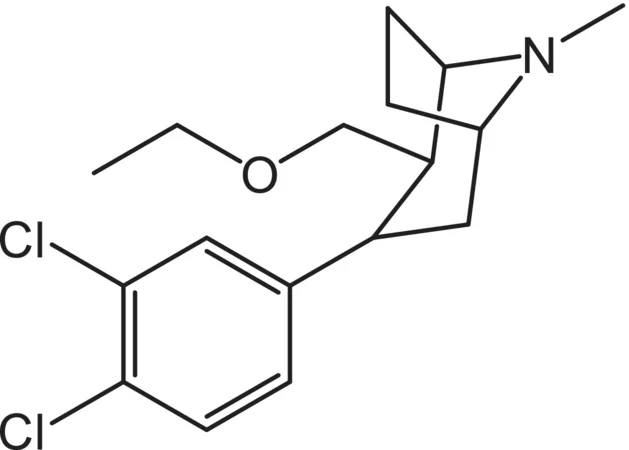
Structure of tesofensine.

Tesofensine (1R,2R,3S,5S)‐3‐(3,4‐dichlorophenyl)‐2‐(ethoxymethyl)‐8‐methyl‐8‐azabicyclo[3.2.1]octane; NS‐2330—shown in Figure 1—is a centrally acting triple monoamine reuptake inhibitor that blocks the presynaptic reuptake of dopamine, norepinephrine, and serotonin [1, 2]. Initially developed for neurodegenerative disorders such as Alzheimer's disease and Parkinson's disease, the compound demonstrated marked weight‐reducing effects in clinical trials and was subsequently repositioned as a potential anti‐obesity drug [3, 4, 5]. In a randomized, double‐blind, placebo‐controlled phase II trial, tesofensine produced dose‐dependent body weight reductions of up to 10%–12% within 24 weeks [4]. Preclinical investigations corroborated these findings, showing reduced food intake and sustained weight loss in obese animal models [6]. Mechanistically, enhanced monoaminergic neurotransmission modulates hypothalamic appetite‐regulating circuits, thereby decreasing caloric intake and altering energy balance [1, 2, 6].

Despite the absence of full marketing authorization [1], tesofensine‐containing products are commercially available as internet‐sold dietary supplements. Owing to its central stimulant properties and pronounced effects on body weight, tesofensine is of particular relevance in competitive sport, especially in weight‐class disciplines and sports where the power‐to‐weight ratio is decisive. Accordingly, the World Anti‐Doping Agency (WADA) classified tesofensine in section S6 (stimulants) of the Prohibited List, banning its use in competition [7]. The emergence of such substances outside regulated pharmaceutical frameworks poses analytical challenges for anti‐doping laboratories, necessitating characterization of elimination profiles and metabolic markers.

While clinical research has primarily addressed efficacy and safety [3, 4, 8], data describing in vivo metabolism are scarce. Published reports primarily mention the intact drug and one dealkylated metabolite as main blood and urinary analytes, and the drug exhibited a plasma half‐life in excess of 200 h [9, 10]. Until today, detailed structural elucidation and systematic investigation of additional phase I and phase II metabolites are lacking. A predominant hepatic metabolism involving cytochrome P450‐mediated oxidative reactions, including hydroxylation and N‐dealkylation, followed by potential phase II conjugation with glucuronic acid or sulfate to enhance renal excretion is likely [9, 11]. However, the relative abundance of free and conjugated metabolites, their excretion profiles, and thus their suitability as urinary markers have not been systematically evaluated in humans.

From an analytical perspective, the identification of specific metabolites is critical to extend detection windows and ensure confirmatory analysis. High‐resolution mass spectrometry (HRMS) enables comprehensive metabolite profiling, accurate mass‐based formula assignment, and retrospective data evaluation [12, 13, 14]. Knowledge of metabolite dissociation behavior, conjugation status, and elimination profiles supports analytical target selection, sample preparation strategies, and the ability to optimize test method sensitivity, all of which are core aspects of anti‐doping analytical workflows [15].

Therefore, the present study aimed to characterize the human metabolism and urinary elimination of tesofensine. In vitro metabolism investigations employing pooled human liver microsomes and S9 fractions were conducted to generate preliminary metabolite candidates and elucidate phase I and phase II biotransformation pathways. Subsequently, a controlled administration study was performed in six healthy volunteers following ingestion of a single 483 μg oral dose via a commercially available supplement. Urine samples were collected up to 600 h to determine metabolite profiles, conjugation patterns, and elimination kinetics.

## 2 Material and Methods

### 2.1 Chemicals and Reagents

Reference material for tesofensine was purchased from Hycultec (Beutelsbach, Germany), and a tesofensine containing product was obtained from an internet‐based supplier. D_3_‐testosterone and acetonitrile (ACN) were obtained from VWR (Darmstadt, Germany). Formic acid (FA), disodium hydrogen phosphate, sodium dihydrogen phosphate, and magnesium chloride were purchased from Carl Roth GmbH (Karlsruhe, Germany). Methanol (MeOH), nicotinamide adenine dinucleotide phosphate (NADPH) tetrasodium salt, uridine 5´‐diphosphate glucuronic acid (UDPGA) trisodium salt, 3´‐phosphoadenosine‐5´‐phosphosulfate (PAPS), D‐saccharic acid 1,4‐lactone monohydrate (SL) were purchased from Merck (Darmstadt, Germany). All chemicals and reagents used were of analytical grade. Deionized water (H_2_O) was produced with a Thermo Scientific^TM^ Barnstead GenPure xCAD Plus system. Solid phase extraction (SPE) Oasis HLB‐cartridges (3mL; 60mg) were purchased from Waters (Eschborn, Germany). Human liver microsomal and S9 fraction enzymatic proteins were obtained from Xenotech (Lenexa, KS).

### 2.2 In Vitro Metabolic Biotransformation of Tesofensine

The in vitro metabolism was conducted in accordance with Kuuranne et al. [16]. The aqueous incubation buffer consisting of 25 mM Na_2_HPO_4_, 14 mM NaH_2_PO_4_, and 1.3 mM MgCl_2_ and an aqueous solution (1 mM) of tesofensine were prepared. NADPH, UDPGA, and saccharic acid lactone solutions (all 50 mM in incubation buffer) were prepared just before adding them to the assay. PAPS (200 μM) and enzymes (20 mg/mL) were aliquoted and frozen at −80°C. Optimized conditions included 20 μM substrate, 18 h of incubation time for phase I at 37°C, and mixing at 450 rpm in an Eppendorf ThermoMixer F1.5, followed by the addition of phase II agents, a further 5 h of incubation at 37°C, and mixing. The total volume for phase I reactions was 50 μL, including 29 μL of incubation buffer, 1 μL of substrate solution, 10 μL of NADPH solution, 5 μL of microsomal enzymes, and 5 μL of S9 fraction enzymes. Preliminary experiments using incubation times of 2, 4, and 6 h were conducted; however, these did not yield sufficient amounts of phase I metabolites. Consequently, a relatively long incubation time of 18 h was selected for the phase I in vitro metabolism approach. Under the applied conditions, no relevant indications of compound instability or nonenzymatic degradation were observed, as confirmed by the absence of corresponding degradation products in control experiments without enzymes. For phase II metabolism experiments, another 50 μL—composed of 10 μL each of UDPGA, PAPS, NADPH, and saccharic acid lactone solutions plus 5 μL of each enzymatic fraction—was added. After a second incubation period of 5 h, the metabolic reaction was stopped through the addition of 150 μL of ice‐cold ACN. The precipitate was removed by centrifugation (2 min; 17,000 x *g*), and the supernatant was transferred to a fresh tube, dried using a vacuum centrifuge, and reconstituted in 0.1% aqueous FA/ACN (99:1; v/v). Each assay included samples with reaction mixtures of (a) all agents, (b) all agents excluding substrate, and (c) all agents excluding enzymes to monitor and differentiate possible enzymatic and nonenzymatic reactions.

### 2.3 In Vivo Metabolites of Tesofensine

The metabolism and elimination profiles of tesofensine and its metabolites in urine were investigated in six healthy male and female volunteers aged from 27 to 51 years after approval by the local ethics committee of the German Sport University Cologne (#120/2024), and informed consent from all participants. The volunteers ingested a single dose of tesofensine in the form of a dietary supplement. The supplement was labelled to contain 500 μg of tesofensine per capsule, which corresponded to a recommended daily dose. Urine samples were collected as described in Section 2.5.

### 2.4 Product Testing

The quantification of tesofensine in the nutritional supplement (labelled to contain 500 μg/capsule) was performed using a standard addition method. A representative sample of the content of one capsule was extracted with 10 mL of 0.1% aqueous FA/ACN (50:50; v/v) and subsequently diluted by a factor of 10,000 to yield a solution of approximately 5 ng/mL. Six 1‐mL aliquots were then fortified with 0, 2, 4, 6, 8, and 10 ng of the dissolved reference material to generate calibration samples containing 0 to 200% of the estimated drug content, in addition. The resulting calibration curve was used to determine the drug content of the supplement used for the administration study described below.

### 2.5 Sample Collection

Six study participants (three male and three female) were recruited. Each participant administered a single dose of the dietary supplement containing 483 μg of tesofensine. Urine samples were collected according to the following schedule: 0 h, ca. 2 h, 4 h, 8 h, 12 h, 24 h, and 48 h post‐administration. Following this 48 h collection period, spot urine samples were collected every 2–3 days (up to 659 h, ca. 28 days). Upon collection, all samples were stored at −20°C until analysis.

### 2.6 Urine Sample Preparation

The urine sample preparation was conducted by solid‐phase extraction (SPE) with Oasis HLB cartridges (3 mL; 60 mg). After SPE cartridge conditioning with 1 mL of methanol and 1 mL of H_2_O, the urine samples (2 mL) were fortified with 10 μL of internal standard (D_3_‐testosterone; 1 μg/mL) and loaded onto the sorbent. The samples were washed with 1 mL of H_2_O and the analytes were eluted with 1 mL of MeOH. Following evaporation to dryness at 50°C under nitrogen flow, the residues were reconstituted in 100 μL of H_2_O/ACN (50:50; v/v) for subsequent LC‐HRMS/MS analysis.

### 2.7 Liquid Chromatography‐Mass Spectrometry

LC‐HRMS/MS analyses of the target analytes were conducted using a Vanquish UHPLC chromatograph coupled via a heated electrospray ionization (HESI) source (300°C, 3 kV, in positive mode) to a Thermo Scientific Exploris 480 mass spectrometer. The chromatographic separation was achieved using a Poroshell 120 EC‐C18 analytical column (50 × 3 mm; 2.7 μm particle size; Agilent, Waldbronn, Germany). The mobile phase was 0.1% FA (aq.) (solvent A) and 0.1% FA in ACN (solvent B). The gradient started at 100% A and decreased to 0% A over 5 min. The column was then flushed at 0% A for 1 min and subsequently re‐equilibrated at 100% A for 4 min. The total run time was 10 min. The flow rate was set to 0.3 mL/min; the injection volume was 5 μL.

The analysis was conducted using targeted MS/MS mode with collision energies (CEs) of 55 and 25 and precursor isolation windows of 1.3 *m/z* (Parallel Reaction Monitoring; PRM). Furthermore, full scan data were acquired in a range of *m/z* 150–1500. Resolutions of 30,000 and 45,000 full width at half maximum (FWHM) at *m/z* 200 in PRM and full‐scan mode were used, respectively.

### 2.8 Method Validation

The LC–MS method for detection and quantification of tesofensine was validated according to current WADA guidelines [17, 18]; the following parameters were determined:

#### 2.8.1 Selectivity

Selectivity was determined by analyzing 10 different blank urine samples (5 male and 5 female samples) to probe for interfering peaks in the selected extracted ion chromatograms at the expected retention times.

#### 2.8.2 Reliability

The reliability was investigated under repeatability conditions by analyzing seven different urine samples fortified with 1 ng/mL of tesofensine, which corresponds to 2% of the WADA minimum required performance level (MRPL) defined for stimulants.

#### 2.8.3 Limit of Detection (LOD) and Limit of Identification (LOI)

The LOD and LOI were estimated by analyzing five batches of 7 different samples fortified with increasing concentrations of tesofensine as follows: 0, 5, 10, 50, 100 pg./mL. Based on the resulting data, the LOD was defined as the lowest concentration at which the most intense product ion at *m/z* 282 was detected at a rate of 95%. By contrast, the two most intense product ions at *m/z* 282 and 110 were evaluated to estimate the LOI (detection rate: ≥ 95%).

#### 2.8.4 Matrix Effects

The ion suppression/enhancement effects were evaluated by comparing the areas of ion transition *m/z* 328–282 of two fortified urine samples and two fortified aqueous samples at concentrations of 5 and 50 ng/mL, each. The duplicate determination was conducted by applying the sample preparation protocol described above. The ratio of the areas of the base peak ion transition *m/z* 328–282 from the matrix sample and the aqueous sample was determined and expressed as a negative percentage (ion suppression). The mean value of both determinations was calculated for both concentration levels.

#### 2.8.5 Carryover

Carryover was assessed by measuring a blank specimen directly after a sample containing 50 ng/mL of the analyte.

#### 2.8.6 Linearity

In urine, the linearity of tesofensine was investigated at concentrations of 0.05, 0.1, 0.5, 1, 5, 10, and 50 ng/mL. Absolute peak areas were used to construct a calibration curve and determine linearity by unweighted regression analysis.

#### 2.8.7 Recovery

To estimate the method's recovery, six urine samples fortified with 10 ng/mL of tesofensine were prepared as described above and compared to six blank sample extracts spiked with the same analyte concentrations prior to LC‐HRMS/MS analysis.

#### 2.8.8 Precision

To assess the intra and interday imprecision of the assay, 3 × 6 samples fortified with three different concentrations of tesofensine (0.1, 1.0, and 10 ng/mL) were analyzed on three consecutive days. Then, the imprecision was assessed by calculating the variation in absolute peak areas and reported as coefficient of variation (CV).

#### 2.8.9 Stability

The stability of the analytes was assessed by repeatedly analyzing the working solutions stored at 4°C during a period of 2 months. Additionally, instrument stability was evaluated by reanalyzing the extracts of validation samples before and after a minimum of 14 days of storage at 10°C in the autosampler.

## 3 Results and Discussion

### 3.1 In Vitro and In Vivo Tesofensine Metabolism Studies

**FIGURE 2:**
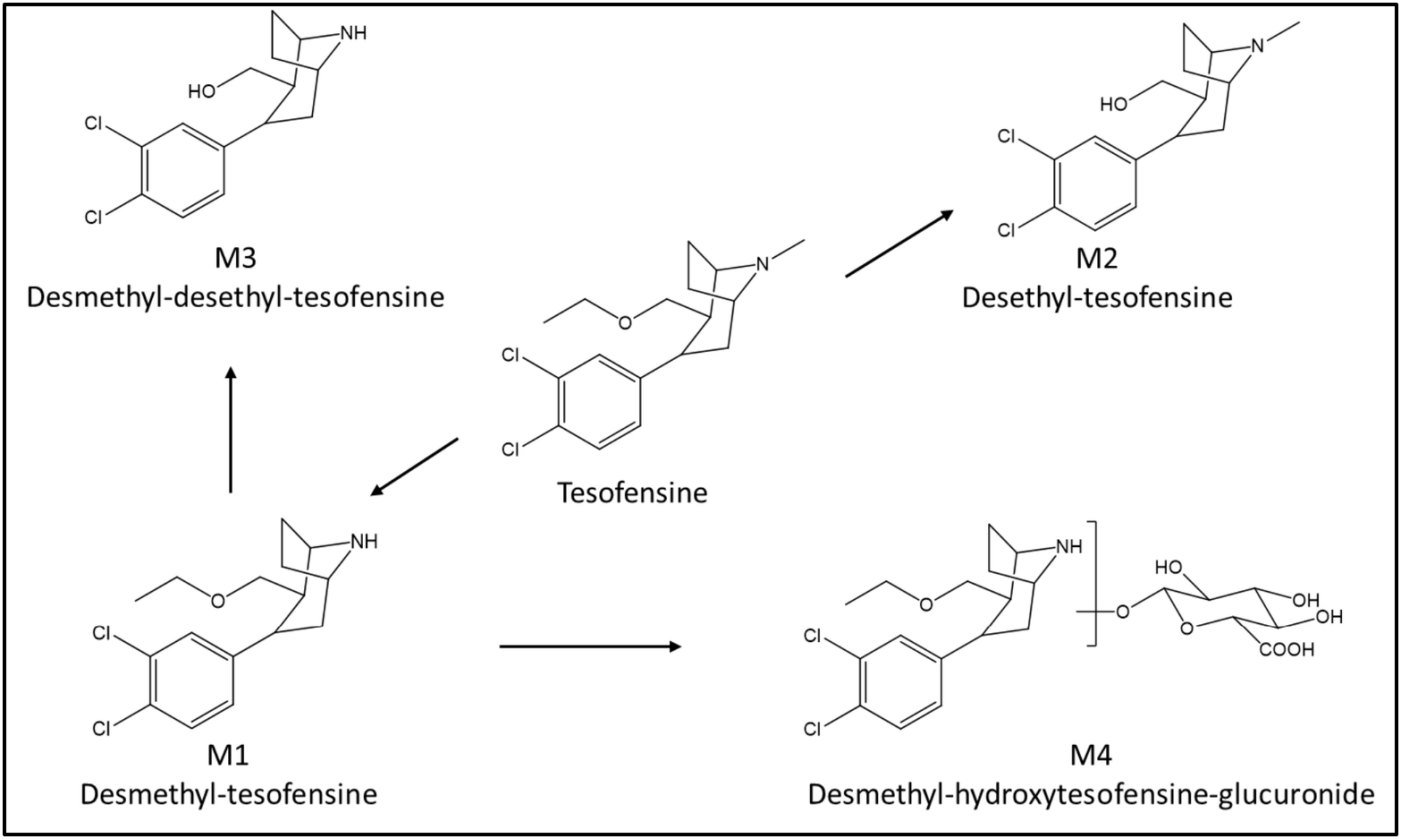
Tesofensine and tentatively assigned tesofensine metabolites M1 to M4.

**FIGURE 3:**
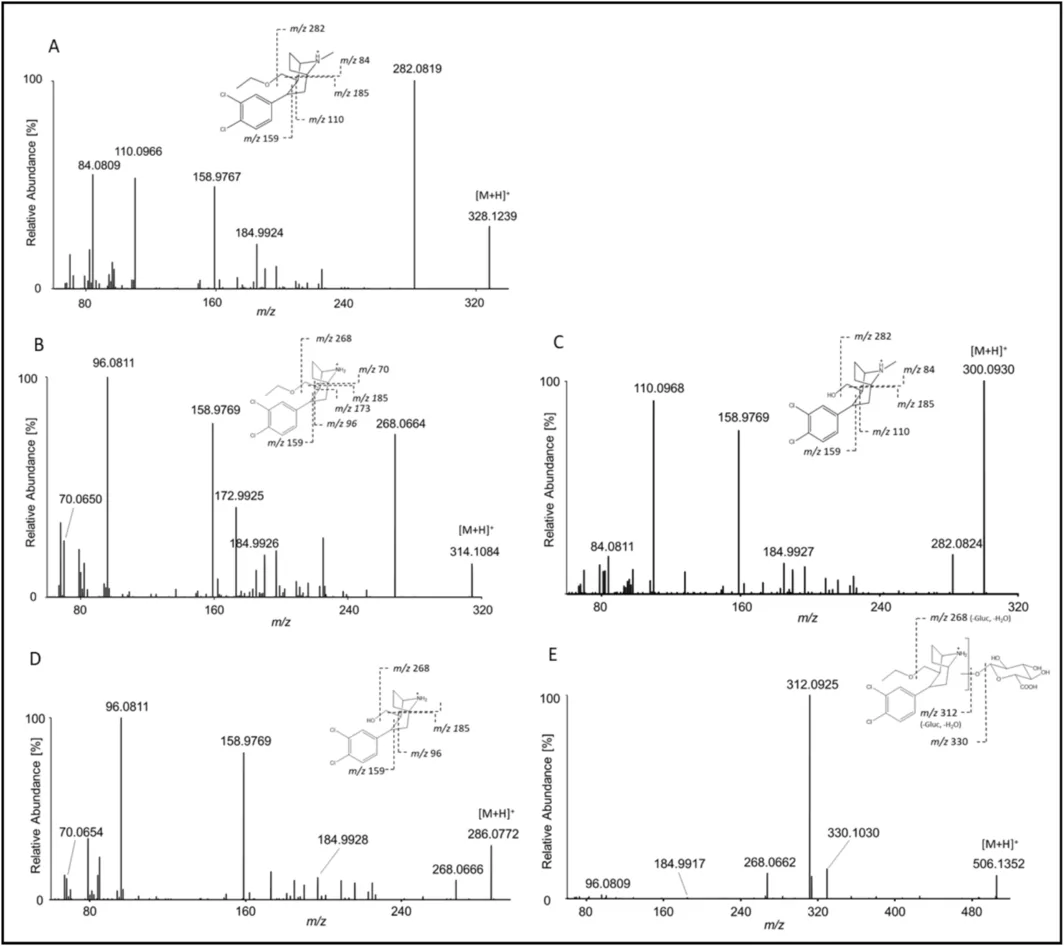
Product ion mass spectra of tesofensine (A), desmethyl‐tesofensine (B), desethyl‐tesofensine (C), desethyl‐desmethyl‐tesofensine (D), and desmethyl‐hydroxytesofensine‐glucuronide (E).

**FIGURE 4:**
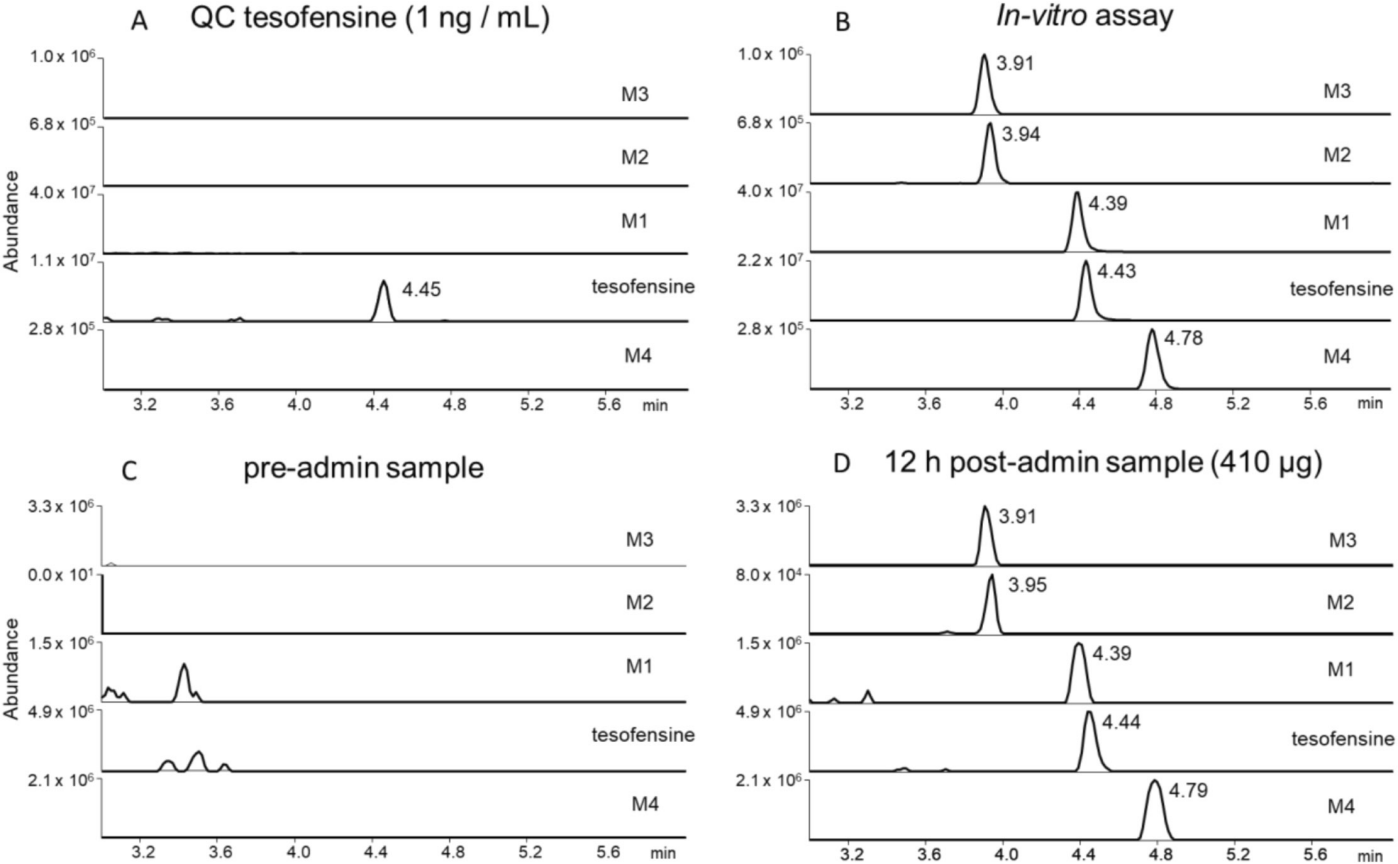
Extracted ion chromatograms of tesofensine and metabolites M1–M4; derived from full‐scan HRMS data: 1 ng/mL tesofensine QC (A), an in vitro incubation assay specimen (B), a blank urine sample (C), and a 12 h post‐administration urine sample (D).

The in vitro metabolism experiments yielded three phase I and one phase II tesofensine metabolites (Figure 2), and no artifacts potentially generated by nonenzymatic reactions were observed. For the identification of metabolic products, product ion mass spectra were recorded after detection of newly formed compounds in full‐scan analyses. Supporting evidence enabling further characterization of these compounds was obtained by high‐resolution/high‐accuracy MS data of precursor and diagnostic product ions (Figure 3), the elemental compositions of which are summarized in Table 1. The observed and characterized in vitro metabolites are in close agreement with those from in vivo experiments (Figure 4).

**TABLE 1:** Experimental elemental composition of tesofensine and metabolites.

Compound retention time	Diagnostic ions [*m/z*]	Elemental composition	Precursor/product ions *m/z* (theor.) *m/z* (exp.)		Error (ppm)
Tesofensine	328	C_17_H_24_Cl_2_NO	328.1229	328.1239	3.0
4.43 min	282	C_15_H_18_Cl_2_N	282.0811	282.0819	2.8
	185	C_9_H_7_Cl_2_	184.9919	184.9924	2.7
	159	C_7_H_5_Cl_2_	158.9763	158.9767	2.5
	110	C_7_H_12_N	110.0964	110.0966	1.8
	84	C_5_H_10_N	84.0808	84.0809	1.2
M1 N‐desmethyl‐tesofensine	314	C_16_H_22_Cl_2_NO	314.1073	314.1084	3.5
4.39 min	268	C_14_H_16_Cl_2_N	268.0654	268.0664	3.7
	185	C_9_H_7_Cl_2_	184.9919	184.9926	3.8
	173	C_8_H_7_Cl_2_	172.9919	172.9925	3.5
	159	C_7_H_5_Cl_2_	158.9763	158.9769	3.8
	96	C_6_H_10_N	96.0808	96.0811	3.1
	70	C_4_H_8_N	70.0651	70.0650	−1.4
M2 Desethyl‐tesofensine	300	C_15_H_20_Cl_2_NO	300.0916	300.0930	4.7
3.94 min	282	C_15_H_18_Cl_2_N	282.0811	282.0824	4.6
	185	C_9_H_7_Cl_2_	184.9919	184.9927	4.3
	159	C_7_H_5_Cl_2_	158.9763	158.9769	3.8
	110	C_7_H_12_N	110.0964	110.0968	3.6
	84	C_5_H_10_N	84.0808	84.0811	3.6
M3 Desethyl‐N‐desmethyl‐tesofensine	286	C_14_H_18_Cl_2_NO	286.0760	286.0772	4.2
3.91 min	268	C_14_H_16_Cl_2_N	268.0654	268.0666	4.5
	185	C_9_H_7_Cl_2_	184.9919	184.9928	4.9
	159	C_7_H_5_Cl_2_	158.9763	158.9769	3.8
	96	C_6_H_10_N	96.0808	96.0811	3.1
	70	C_4_H_8_N	70.0651	70.0654	4.3
M4 N‐desmethyl‐hydroxytesofensine‐glucuronide	506	C_22_H_30_Cl_2_NO_8_	506.1343	506.1352	1.8
4.78 min	330	C_16_H_22_Cl_2_NO_2_	330.1022	330.1030	2.4
	312	C_16_H_20_Cl_2_NO	312.0916	312.0925	2.9
	268	C_14_H_16_Cl_2_N	268.0654	268.0662	3.0
	185	C_9_H_7_Cl_2_	184.9919	184.9917	1.1
	96	C_6_H_10_N	96.0808	96.0809	1.0

### 3.2 Mass Spectrometric Characterization of Tesofensine and Metabolites

Structurally, tesofensine and its metabolites are dichlorophenyl‐substituted tertiary amines. Under positive ESI conditions, tesofensine forms a stable protonated molecular ion at *m/z* 328 ([M + H]^+^) and, together with its metabolites, shows ion species with a characteristic diagnostic chlorine isotope cluster in full‐scan mass spectra. The dissociation pattern is dominated by a cleavage attributable to the elimination of the ethoxy group. This cleavage yields a prominent product ion at *m/z* 282, corresponding to the loss of a neutral fragment of 46 Da (ethanol). A second highly abundant and diagnostically important product ion is found at *m/z* 185 (C_9_H_7_Cl_2_^+^), representing a dichlorobenzyl‐type cation. This product ion is suggested to originate from benzylic bond cleavage between the aromatic ring and the aliphatic side chain. The observed abundance of this product ion may be attributed to the overall stability of the dichlorinated aromatic system and favorable fragmentation behavior under the applied collision‐induced dissociation conditions. Subsequent dissociation is proposed to yield a product ion at *m/z* 159 (C_7_H_5_Cl_2_^+^), via loss of C_2_H_2_. The persistence of these dichlorinated aromatic ions across all investigated phase I metabolites confirms that biotransformation occurs primarily at the amine moiety rather than on the aromatic ring.

Phase I metabolism of tesofensine is dominated by dealkylation reactions potentially mediated by cytochrome P450 enzymes, leading to N‐desmethyl‐ (M1) and desethyl‐tesofensine (M2), as well as the combined desethyl‐N‐desmethyl metabolite (M3). In MS/MS spectra, these metabolites exhibit systematic 14 Da mass shifts reflecting metabolic desmethylation and desethylation reactions. Their dissociation pathways are mechanistically analogous to that observed for the intact drug. For example, M1 ([M + H]^+^ at *m/z* 314) formed a major product ion at *m/z* 268, while retaining the characteristic aromatic ions at *m/z* 185 and 159. Similarly, M2 ([M + H]^+^ at *m/z* 300) and M3 ([M + H]^+^ at *m/z* 286) exhibited convergent fragmentation patterns, indicating that N‐dealkylation does not alter the intrinsic dissociation pathways but only shifts the masses of nitrogen‐containing ions.

Lower‐mass product ions such as those at *m/z* 110, 96, 84, and 70 correspond to aliphatic iminium species formed by cleavage within the amine‐containing side chain. The exact *m/z* values of these ions vary depending on the degree of alkyl substitution, further supporting structural assignments of the metabolites.

In addition to phase I metabolites, tesofensine undergoes phase II conjugation, notably O‐glucuronidation following hydroxylation. However, no corresponding hydroxylated phase I metabolite could be observed under selected experimental conditions. The desmethylated and hydroxylated tesofensine glucuronide (M4) exhibits a protonated molecular ion at *m/z* 506. The dissociation pathway involves the neutral loss of 176 Da, corresponding to cleavage of the glucuronic acid moiety [19], and the resulting aglycone subsequently featured the loss of 18 Da (H_2_O) as well as the same cleavage and aromatic bond dissociation reactions observed for the N‐demethylated phase I metabolites, again producing the ions *m/z* 268 and *m/z* 96 (Figure 3E).

The dissociation behavior of tesofensine and its metabolites is advantageous for doping control purposes. The conserved formation of the dichlorobenzyl cation at *m/z* 185 and other plausible product ions provides robust confirmatory transitions for targeted LC‐HRMS or LC‐MS/MS methods. Moreover, the systematic mass differences between parent drug and metabolites enable clear differentiation of dealkylated species while preserving structural coherence across the metabolic series. Together, these features allow sensitive and specific detection of tesofensine misuse in biological matrices.

### 3.3 LC‐HRMS/MS Analysis of Tesofensine and Metabolites

Figure 4 shows the extracted ion chromatograms (EICs) of tesofensine and its metabolites M1–M4 derived from full‐scan HRMS data under reversed‐phase LC conditions. In the quality control (QC) sample containing 1 ng/mL tesofensine (Figure 4A), a distinct peak corresponding to the parent compound was observed at a retention time of 4.45 min. No signals were detected at the retention times of the investigated analytes in the blank sample (Figure 4C), demonstrating the selectivity of the analytical method and the absence of interfering matrix components.

For the in vitro assay (Figure 4B), several metabolite signals were detected in addition to the parent compound. The metabolites M2 and M3 eluted at earlier retention times (approximately 3.9 min), indicating a higher polarity compared with tesofensine. Metabolite M1 eluted slightly before the parent compound (4.39 min), whereas metabolite M4 showed the longest retention time (4.78 min). A comparable chromatographic pattern was observed for the urine sample collected 12 h after administration (Figure 4D), where tesofensine and all investigated metabolites (M1–M4) were detected at retention times consistent with those observed in the in vitro assay. The presence of the same metabolites in both the in vitro experiment and the post‐administration sample supports the relevance of the in vitro metabolic model for predicting the metabolic profile observed in vivo.

### 3.4 Validation Results

The validation results for the developed method are summarized in Table 2. The method for urine sample analysis was found to be selective and no interfering signal was observed at the expected retention time of tesofensine. The reliability was successfully demonstrated at a concentration of 1 ng/mL. Detection and identification limits for tesofensine were estimated at 0.01 ng/mL. Matrix effects for tesofensine were 81%. At a concentration of 50 ng/mL, a carryover of 0.4% could be observed and the method was found to be linear from 0.05 to 10 ng/mL (*R* ^2^ = 0.997). The recovery rate was 34% at a concentration of 10 ng/mL. The intra and interday imprecisions were investigated at concentrations of 0.1, 1, and 10 ng/mL and yielded coefficients of variation between 2.6% and 17.8% for intraday imprecision and between 5.8% and 20.4% for interday imprecision, respectively. Stability testing showed that both the standard solutions stored for 2 months at 4°C and the sample extracts kept in the autosampler for at least 14 days at 10°C were stable.

**TABLE 2:** Validation results of the quantitative method for the determination of tesofensine in urine.

Validation parameter	Concentration(s) [ng/mL]	Result
Selectivity	—	0/10
Reliability	1	7/7
Matrix effect	5 and 50	−81%
Linearity	0.05–10	*R* ^2^ = 0.997
Recovery	10	34%
LOD	0.005–0.1	0.01 ng/ml
LOI	0.005–0.1	0.01 ng/ml
Intraday‐imprecision	0.1	9.2%–17.8%
	1	5.1%–5.9%
	10	2.6%–3.4%
Interday‐imprecision	0.1	20.4%
	1	8.2%
	10	5.8%

### 3.5 Urinary Elimination of Tesofensine and Its Metabolites

The conducted excretion study was designed to characterize the urinary elimination profile of tesofensine and its metabolites following controlled oral administration. After determination of baseline conditions, a single dose of 483 μg tesofensine in capsule form (as determined by product analysis prior to drug administration) was administered to the participants. The time of intake was recorded as time zero (t = 0).

Urine samples were collected longitudinally over multiple time intervals to obtain a comprehensive excretion profile. Typically, these intervals included short collection periods during the first hours post‐administration (e.g., 0–2 h, 2–4 h, 4–8 h), followed by extended intervals (12–24 h), and additional collections on subsequent days (e.g., 24–48 h, 48–72 h).

Tesofensine and its metabolites (M1–M4) showed distinct temporal patterns in terms of maximum concentration, time to peak, and detection windows. These differences provide insight into the metabolic processing of the parent compound and are relevant for analytical detection, particularly in anti‐doping contexts.

**FIGURE 5:**
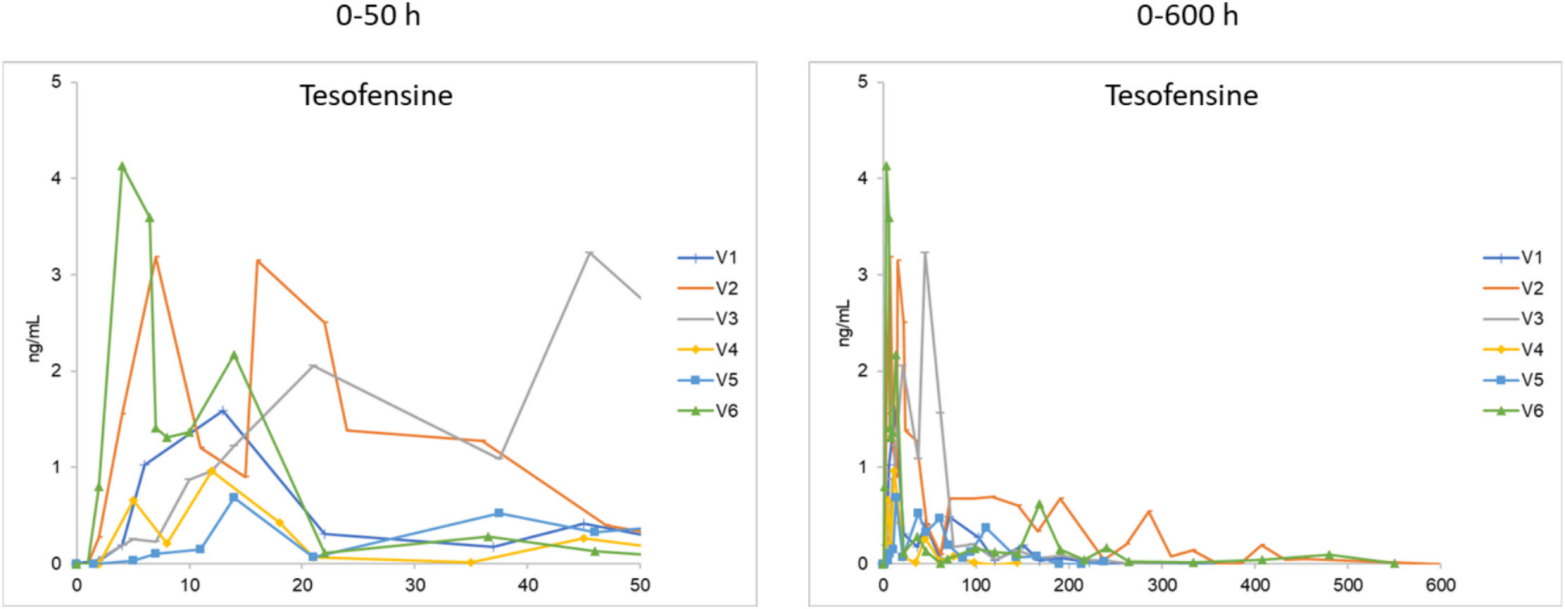
Elimination profile of tesofensine after a single oral dose of 483 μg tesofensine in six volunteers. Left: first 50 h zoom‐in; right: full timeline.

The urinary elimination profile of tesofensine (Figure 5) shows maximum concentrations, ranging from 0.7 to 4.2 ng/mL. Peak concentrations were reached between 4 and 48 h after administration. Thereafter, the levels gradually declined and returned to baseline between 150 and 550 h. The relatively broad elimination window indicates interindividual variability in the metabolism and excretion of the parent compound. However, compared with some of the metabolites, tesofensine itself appears to provide a long but not the longest detection window.

**FIGURE 6:**
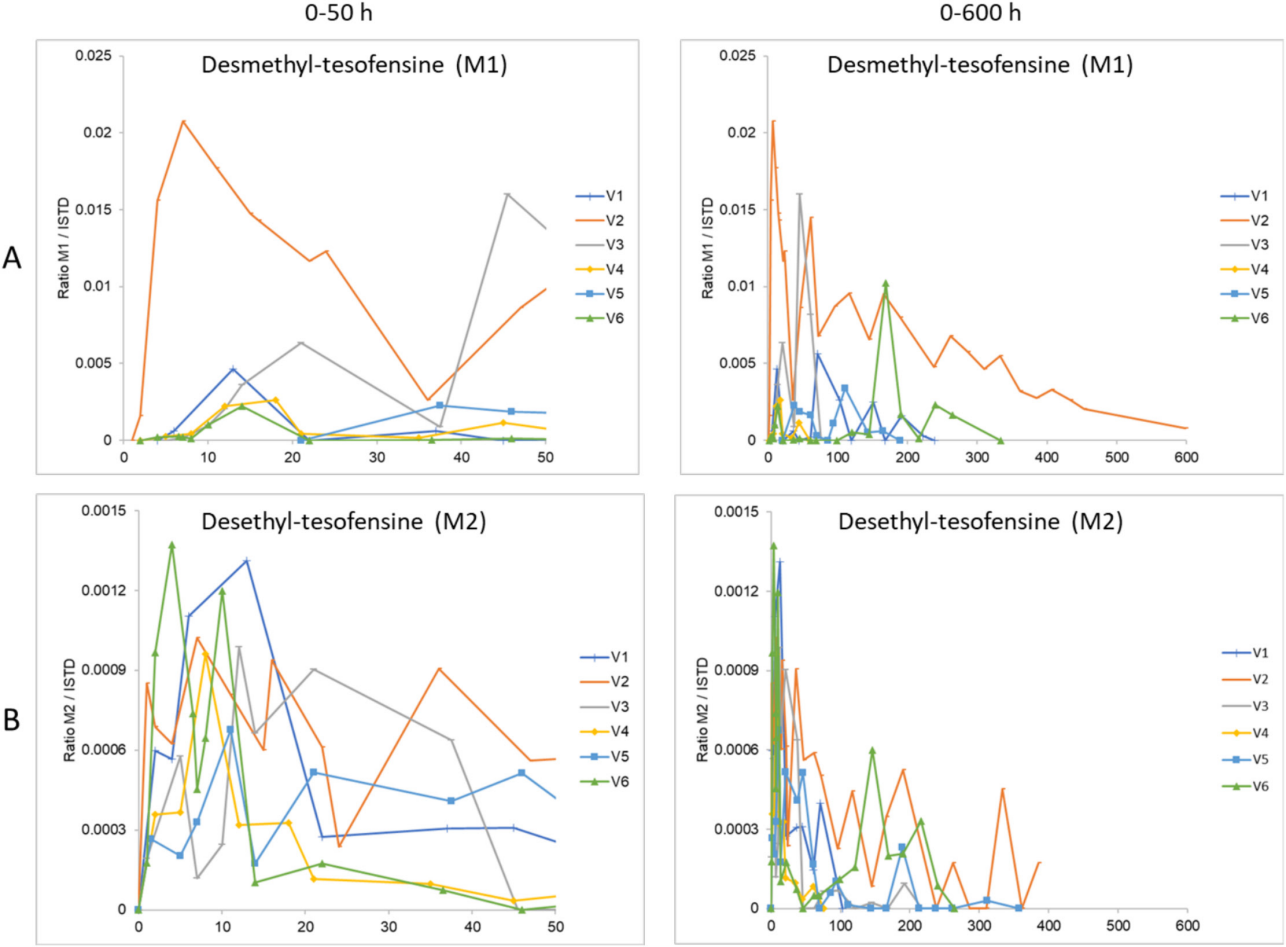
Elimination profiles of desmethyl‐tesofensine (A) and desethyl‐tesofensine (B) after a single oral dose of 483 μg tesofensine in six volunteers. Left: first 50 h zoom‐in; right: full timeline.

For metabolites M1‐M4, quantitative concentrations could not be determined because certified reference material was unavailable; therefore, the ratio of analyte peak area to internal standard peak area was used. M1 shows maximum signal intensities between 6 and 150 h after administration (Figure 6A). The decline to baseline occurs between 75 h and more than 600 h. In some subjects, M1 may persist for very long periods, making it potentially useful as a long‐term marker. M2 exhibits comparatively early peak levels, the maximal values being reached between 4 and 12 h after administration (Figure 6B). However, also here the elimination phase varied substantially among individuals, and detection windows ranged from approximately 75 to more than 380 h. Although M2 appears to be eliminated into urine rapidly after drug administration, its detection window can still extend over several days to weeks depending on the subject.

**FIGURE 7:**
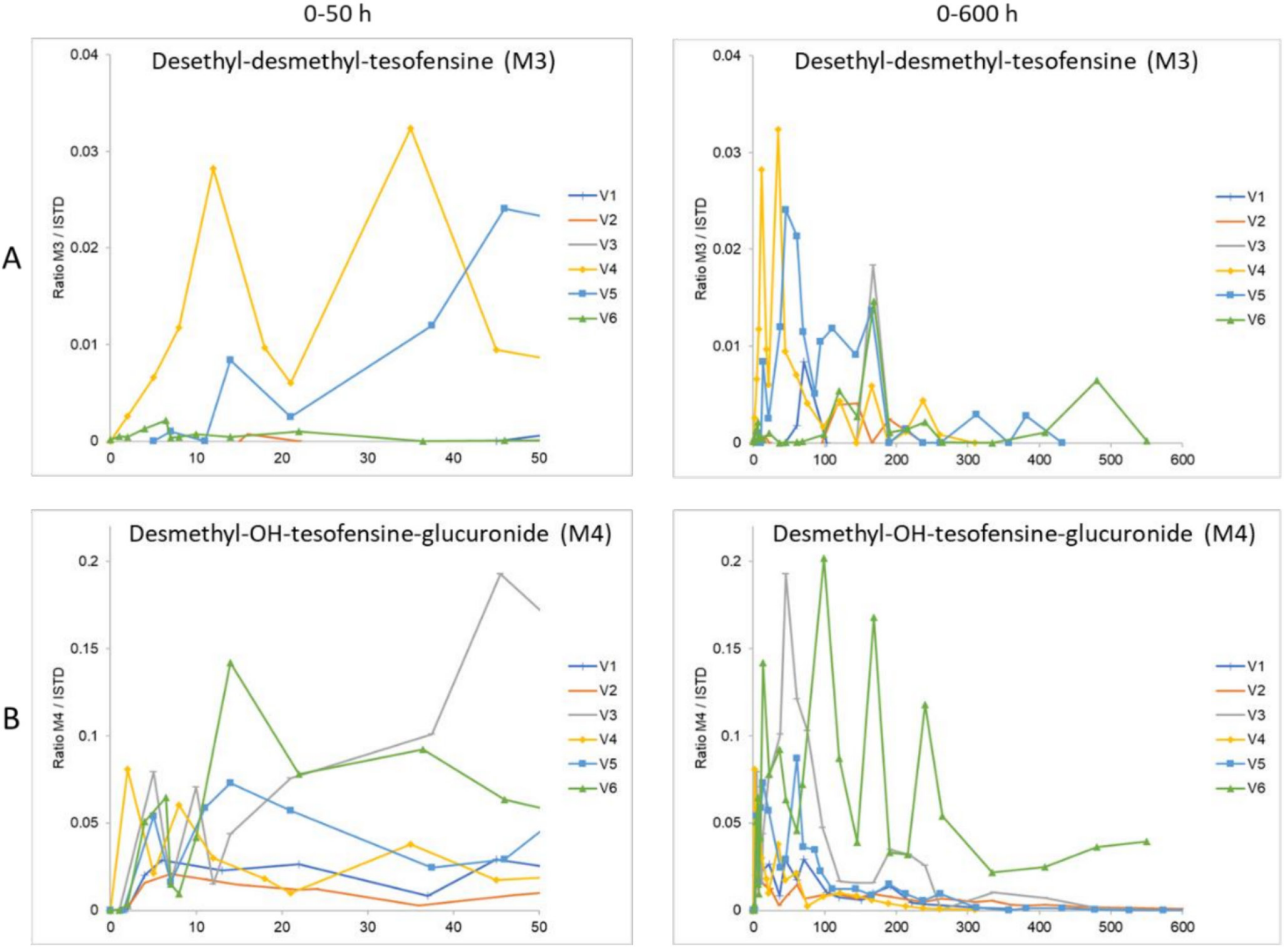
Elimination profiles of desmethyl‐desethyl‐tesofensine (A) and desmethyl‐OH‐tesofensine‐glucuronide (B) after a single oral dose of 483 μg of tesofensine in six volunteers. Left: first 50 h zoom‐in; right: full timeline.

M3 shows a markedly delayed excretion profile compared to the other metabolites. Maximum levels were reached after 35 to 175 h, indicating that it is likely a secondary or downstream metabolite formed later in the metabolic pathway. Correspondingly, the detection window is broad, ranging from about 200 to over 550 h (Figure 7A). Interestingly, higher or clearly detectable levels can be primarily observed in Subjects 4, 5, and 6 (all females). This observation suggests possible sex‐related differences in metabolic pathways or enzyme activity responsible for M3 formation, but further data are required to verify this hypothesis. The excretion profile of M4 (Figure 7B) also features a broad window of maximum urinary levels, ranging from 2 to 100 h after drug administration, and the metabolite was detected until 300 and over 550 h. This extended detection window suggests that M4 may serve as a useful marker for retrospective detection when the parent compound is no longer detectable. Taken together, the excretion profiles demonstrate that tesofensine undergoes extensive metabolism, producing metabolites with markedly different formation and elimination kinetics. Early‐forming metabolites such as M2 appear shortly after administration, whereas metabolites like M3 reach peak levels much later. Several metabolites, particularly M1, M3, and M4, show long detection windows extending beyond 500 h in some individuals. Such long‐term metabolites are especially valuable for analytical purposes because they increase the probability of detecting prior drug intake. Additionally, the observed interindividual variability—potentially influenced by biological factors such as sex—highlights the importance of monitoring multiple metabolites when establishing reliable detection strategies.

## 4 Conclusions

This study provides a comprehensive characterization of the metabolism and urinary elimination of tesofensine following controlled oral administration of a commercially available dietary supplement. In vitro experiments using human liver microsomes and S9 fractions, complemented by an in vivo administration study in six volunteers, enabled the tentative identification of four principal metabolites: three phase I dealkylation products (M1–M3) and one hydroxylated glucuronide conjugate (M4). Structural assignments were supported by high‐resolution MS/MS data and characteristic fragmentation pathways, demonstrating consistent diagnostic ions suitable for confirmatory analysis.

The validated LC‐HRMS/MS method allowed sensitive detection of tesofensine in urine with a limit of detection of 0.01 ng/mL. Following administration of a single 483 μg dose, maximum urinary concentrations of the parent compound ranged from approximately 0.7 to 4.2 ng/mL and were typically observed within 4–48 h. Several metabolites, particularly M1, M3, and M4, exhibited extended detection windows exceeding 500 h in some individuals, highlighting their potential value as complementary analytical targets in doping control. However, interpretations concerning potential anti‐doping rule violations should be made with caution, as tesofensine is classified under substance class S6 (stimulants) and is therefore prohibited in‐competition only.

Considering that the minimum reporting level defined by the World Anti‐Doping Agency for most stimulants is 50 ng/mL, the urinary concentrations observed in this study remain substantially below this value. Consequently, the ingestion of tesofensine‐containing products at the recommended dosage is unlikely to result in concentrations that would constitute an adverse analytical finding. Nevertheless, the prolonged detectability of the parent compound and several metabolites underscores the importance of including these markers in comprehensive screening strategies to enable reliable identification of tesofensine use in sports.

In addition, considering the potential weight‐reducing effects of tesofensine, a future evaluation of the compound in the context of weight‐reducing substances may be of relevance, particularly for sports involving weight categories or those disciplines where power‐to‐weight ratios are critical. The analytical and excretion data presented in this study may therefore contribute to future discussions regarding the anti‐doping relevance and potential monitoring strategies for tesofensine.

## Funding

This project was conducted with support of the Manfred‐Donike Institute for Doping Analysis (Cologne, Germany) and the Federal Ministry of the Interior and Community (Berlin, Germany).

## Conflicts of Interest

The authors declare no conflicts of interest.

## Data Availability

The data that support the findings of this study are available on request from the corresponding author.
